# Output Characterization of 220 nm Broadband 1250 nm Wavelength-Swept Laser for Dynamic Optical Fiber Sensors

**DOI:** 10.3390/s22228867

**Published:** 2022-11-16

**Authors:** Gi Hyen Lee, Soyeon Ahn, Min Su Kim, Sang Won Lee, Ji Su Kim, Byeong Kwon Choi, Srinivas Pagidi, Min Yong Jeon

**Affiliations:** 1 Department of Physics, College of Natural Sciences, Chungnam National University, 99 Daehak-ro, Yuseong-gu, Daejeon 34134, Republic of Korea; 2 Institute of Quantum Systems (IQS), Chungnam National University, 99 Daehak-ro, Yuseong-gu, Daejeon 34134, Republic of Korea

**Keywords:** wavelength-swept laser, fiber lasers, semiconductor optical amplifier, dynamic measurement, dynamic optical fiber sensors

## Abstract

Broadband wavelength-swept lasers (WSLs) are widely used as light sources in biophotonics and optical fiber sensors. Herein, we present a polygonal mirror scanning wavelength filter (PMSWF)-based broadband WSL using two semiconductor optical amplifiers (SOAs) with different center wavelengths as the gain medium. The 10-dB bandwidth of the wavelength scanning range with 3.6 kHz scanning frequency was approximately 223 nm, from 1129 nm to 1352 nm. When the scanning frequency of the WSL was increased, the intensity and bandwidth decreased. The main reason for this is that the laser oscillation time becomes insufficient as the scanning frequency increases. We analyzed the intensity and bandwidth decrease according to the increase in the scanning frequency in the WSL through the concept of saturation limit frequency. In addition, optical alignment is important for realizing broadband WSLs. The optimal condition can be determined by analyzing the beam alignment according to the position of the diffraction grating and the lenses in the PMSWF. This broadband WSL is specially expected to be used as a light source in broadband distributed dynamic FBG fiber-optic sensors.

## 1. Introduction

A wavelength-swept laser (WSL) is a tunable laser in which a narrow-linewidth laser is sequentially and rapidly tunable over a wide wavelength region (>100 nm). WSLs have been typically developed as light sources in optical coherence tomography (OCT) [1,2,3,4,5,6,7,8,9], dynamic fiber-optic sensors [10,11,12,13,14,15,16], and light detection and ranging (LiDAR) [17,18]. WSLs have been applied in various fields owing to the characteristics of a wide scanning range, fast scanning speed, strong intensity, narrow linewidth, and one-to-one correspondence with the same waveform in the spectral and temporal domains.

The rapid scanning speed of WSLs is related to the sensitivity of dynamic fiber optic sensors and the OCT real-time imaging acquisition, and the narrow linewidth is related to the penetration depth of the sample in the OCT system. High-speed WSLs have been implemented in various ways [1,2,3,4,5,6,7,8,9,19,20,21,22,23,24]. Recent studies on high-speed WSLs have mainly focused on increasing the repetition rate of the scanning frequency by using a Fabry-Pérot wavelength tunable filter (FPTF) or MEMS-based mechanical filter, driven at a scanning frequency of several hundred kilohertz, or by using an optical interleaving method. On the other hand, a WSL with a wide scanning range can increase the measurement range in optical fiber sensors and the resolution of OCT images [6,7,9,19,20,21,22,23,24]. In particular, WSLs with a broadband scanning range of 150 nm or more are useful in fiber-optic sensors. According to the results of recent studies, the scanning range of a wideband WSL implemented using a single semiconductor optical amplifier (SOA) is limited to within 150 nm [7,9,13,19,20,21]. WSLs with a wider wavelength scanning range can be implemented by combining two SOAs with different center wavelengths, and have been described in several recent studies [16,22,23,24].

WSLs are implemented using several types of wavelength-scanning filters or by tuning the dispersion of the laser resonator [1,3,4,6,19,25,26]. The FPTF-based WSL has the advantage of being easily implemented without the need for optical alignment, because all devices are pigtailed with optical fibers. However, the FPTF is thermally unstable, and it is cumbersome to replace the filter when the free spectral range (FSR) is changed. Although a polygonal mirror scanning wavelength filter (PMSWF)-based WSL has mechanical limitations and difficulties in beam alignment in free space, the output characteristics of the WSL can be changed by appropriately adjusting the optical parameters of the diffraction grating and lenses.

In this study, we present the characteristics of broadband WSLs over a 220-nm scanning bandwidth by connecting two SOAs in parallel based on a PMSWF. As the WSL scanning speed increases, the output intensity and bandwidth decrease. Therefore, we analyzed this phenomenon as the concept of the saturation limit frequency that accumulates in the amplified spontaneous emission (ASE) background and reaches the saturation power, and we determine experimentally that it is partially relevant. To increase the scanning range and output intensity of the WSL, fine alignment of the polygonal mirrors and accurate optical alignment between the two lenses and the beam incident on the diffraction grating are important. We report the result of obtaining the optimal scanning bandwidth by analyzing the output dependence of the WSL according to the alignment of the optical axis of the diffraction grating and lenses.

## 2. Saturation Limit Frequency

Most WSLs exhibit a decrease in power and bandwidth as the sweeping speed increases. This is due to several factors, such as the gain and loss of the laser, polarization, and nonlinear effects. However, the main cause is that the laser oscillation time becomes insufficient as the sweeping speed increases. The WSL requires the oscillation time to reach saturation power. If this is not satisfied, the laser power decreases exponentially. In a WSL, the oscillation of a specific wavelength that meets the filtering conditions of the tunable wavelength filter is repeated and sequentially performed. Therefore, a single wavelength is characterized by periodic oscillation followed by extinction. As the sweep speed increases, the oscillation period becomes shorter; therefore, the oscillation time becomes insufficient, and the laser power decreases exponentially. In a WSL, the frequency at which the laser builds up from the ASE background to reach saturation power is the saturation limit frequency [3]. In other words, it is related to the maximum sweep speed for the WSL to maintain the saturation output. The output of a wavelength component filtered by the tunable filter is related to the number of roundtrips of the resonator to match the filtering condition. The number *n* of roundtrips required to reach the saturation output is

(1) $$n=\frac{log\left(\frac{P_{\mathrm{sat}}}{P_{\mathrm{ASE}}}\right)}{log\left(\beta \right)},$$

where $P_{\mathrm{sat}}$ is the saturation power, $P_{\mathrm{ASE}}$ is the power of the ASE, and β is the round-trip net gain, which is calculated as the difference between the small-signal gain of the laser medium and the loss per round trip [3]. The time per cavity round-trip $\tau_{roundtrip}$ is

(2) $$\tau_{roundtrip}=\frac{L\cdot n_{ref}}{c}\;,$$

where L is the cavity length, $n_{ref}$ is the refractive index of the laser cavity, and c is the speed of the light. The build-up time to reach the saturation output power using Equations (1) and (2) is as follows:

(3) $$\tau_{buildup}=n\cdot \;\tau_{roundtrip}=\frac{\log\left(\frac{P_{\mathrm{sat}}}{P_{\mathrm{ASE}}}\right)}{log\left(\beta \right)}\frac{L\cdot \;n_{ref}}{c}.$$

The maximum frequency (saturation limit frequency) from the buildup time can be estimated as follows:

(4) $$f_{max}\approx \frac{\Delta \lambda }{\tau_{buildup}\cdot \Delta \lambda_{tunningrange}}\approx \frac{\log\left(\beta \right)\cdot \;c\cdot \;\Delta \lambda }{\log\left(\frac{P_{\mathrm{sat}}}{P_{\mathrm{ASE}}}\right)\cdot \;L\cdot \;n_{ref}\cdot \;\Delta \lambda_{tuningrange}}\;,$$

where Δλ is the linewidth of the tunable filter, and $\Delta \lambda_{tuningrange}$. is the total tuning bandwidth [3]. For the case of the FPTF, the wavelength swept is not performed up to the FSR, which is the maximum tunable range achieved through electrical signal control. However, the PMSWF has an FSR equal to $\Delta \lambda_{tuningrange}$. The saturation limit frequency is the maximum sweep frequency at which the saturation output is maintained. This is one of several factors that decreases the output power with increasing swept frequency, and it is difficult to calculate an exact value. However, because the saturation limit frequency is a major factor in the operation of the WSL, various pieces of information can be obtained from the output analysis according to the sweep frequency. First, it is possible to determine the relationship between the operating variables of the WSL in the output characteristics according to the increase in sweep frequency. Increasing the saturation limit frequency by, for example, increasing β and Δλ or decreasing $\Delta \lambda_{tuningrange}$ and L, mitigates the decrease in optical output power as the sweep frequency increases. Second, the cause of the decrease in scanning bandwidth with an increase in sweep frequency can be identified. The saturation limit frequency is related to the optical output power, but it is also closely related to the scanning bandwidth. This is also related to the decrease in the scanning bandwidth and dependence of the output power on the sweep frequency. 

## 3. Experiments

**Figure 1:**
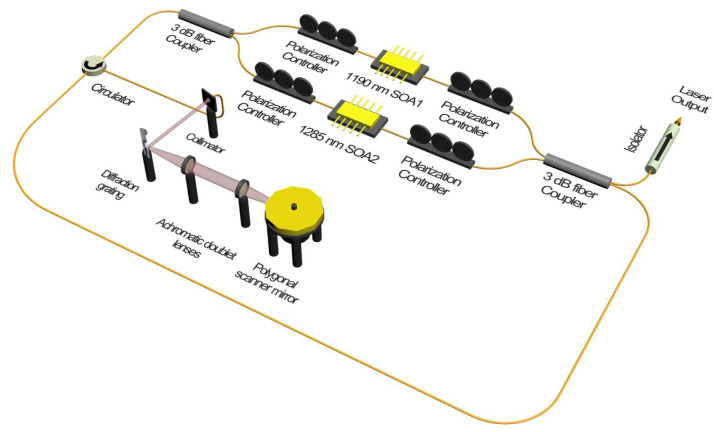
Schematic diagram of experimental setup.

**Figure 2:**
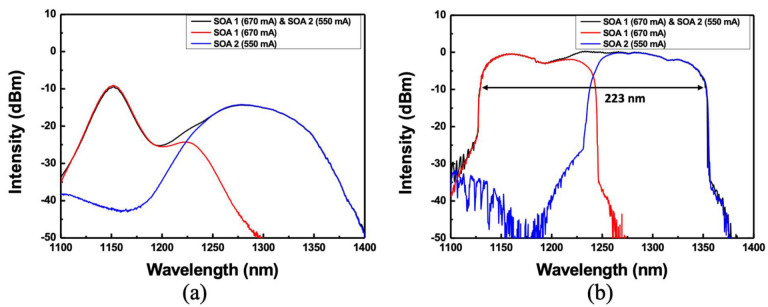
(**a**) ASE spectra of two SOAs, and (**b**) optical spectra from the broadband WSL.

Figure 1 shows a schematic diagram of the experimental setup for broadband WSLs. Two SOAs with different center wavelengths are implemented by connecting them in parallel using a Mach–Zehnder interferometer. The experimental setup consists of two 3 dB fiber couplers, two SOAs, four polarization controllers, an optical circulator, and a PMSWF. The PMSWF is composed of a telescope with two achromatic doublet lenses, a brazed diffraction grating, and a 72-facet polygonal scanner mirror. As the two SOAs have polarization dependence, the polarization controllers in front of and behind the SOA are appropriately adjusted to match the intensity in a wide wavelength band. Even if the two arms of the Mach–Zehnder interferometer are of unequal length, this does not significantly affect the output of the WSL. The optical path length of laser resonator lengths for each path of SOA 1 and SOA 2 including free space are 16.35 m and 18.61 m, respectively. The output from the WSL was monitored using an oscilloscope and an optical spectrum analyzer through an optical isolator. Two SOAs with center wavelengths of 1190 and 1285 nm are used, as shown in Figure 2a. The red line of SOA 1 (Innolume Inc., Dortmund, Germany) has an ASE center wavelength of 1190 nm and the 10-dB bandwidth is 49 nm (from 1131 nm to 1180 nm). In the blue line of SOA 2 (O-Band Booster Optical Amplifier; Thorlabs, Newton, NJ, USA), the center wavelength of the ASE is 1285 nm and the 10-dB bandwidth is 75 nm (from 1249 nm to 1324 nm). The black line represents the combined spectra of the two SOAs. 

**Figure 3:**
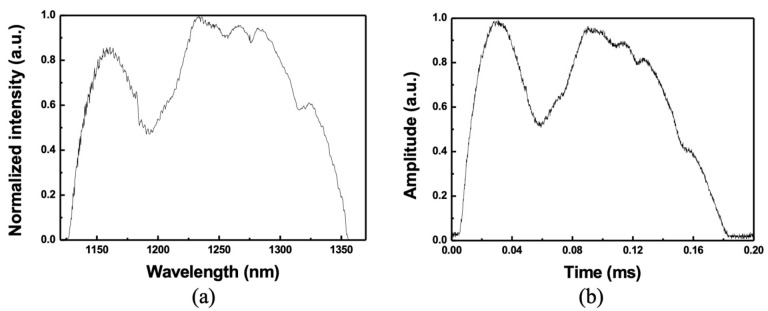
(**a**) Optical spectrum (linear scale), and (**b**) temporal output of wideband WSL.

Figure 2b shows the optical spectra of the broadband WSL. The red and blue lines show the optical spectra when only SOA 1 or SOA 2 is connected to the Mach–Zehnder interferometer, respectively. At a scan rate of 3.6 kHz when only SOA 1 is connected, the 10-dB bandwidth and average output power are ~115 nm (from 1129 nm to 1244 nm) and 4.1 mW, respectively. However, when only SOA 2 is connected, the 10-dB bandwidth and average output power are ~115 nm and 5.81 mW, respectively. When both SOAs are connected at a scanning speed of 3.6 kHz, the optical spectrum is represented by a black line. The 10-dB bandwidth and average optical output power are 223 nm (from 1129 nm to 1352 nm) and 10.1 mW, respectively. Figure 3a,b show the outputs in the spectral and temporal domains of the broadband WSL, respectively. As described above, the shapes are similar and show a one-to-one correspondence with each other. This characteristic enables real-time detection by measuring the response signal of the pulse in the temporal domain, instead of the response in the spectral domain of the optical fiber dynamic sensor. 

For broadband WSLs, the net gain is very important, as it relates to optical alignment. To increase the scanning range and optical output power in the WSL, the fine alignment of the polygonal scanning mirror, perpendicularity between the optical path and lenses, and precise optical alignment between the beam incident on the diffraction grating and two lenses are important. The failure of precise optical alignment due to these factors increases the loss of the laser cavity and limits the scanning range beyond 200 nm. Therefore, we investigated the output dependence of the WSL according to the optical axis alignment of the diffraction grating and lenses.

**Figure 4:**
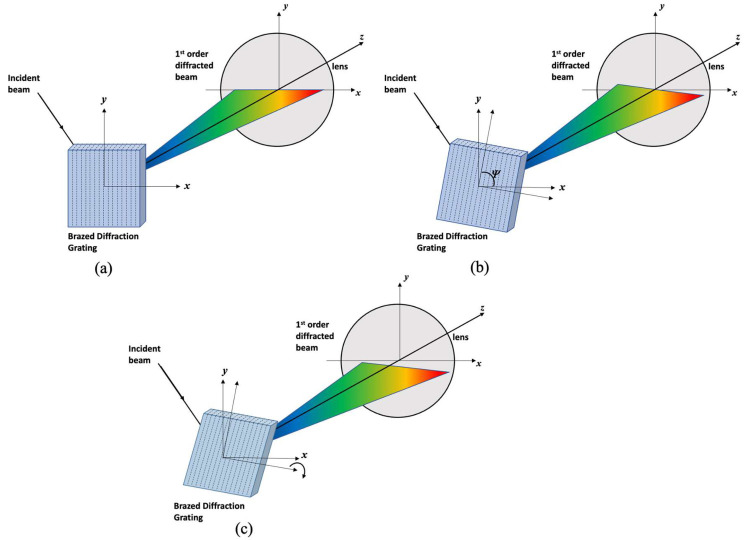
Schematic diagrams of the first-order diffracted beam incident on the lens surface according to the alignment of the diffraction grating; (**a**) ideal alignment, (**b**) the direction of the grooves is slightly rotated around the *z*-axis, and (**c**) in the case of (**b**), it is slightly rotated around the *x*-axis.

Let the axis of the beam traveling direction be denoted by the *z*-axis and the lens surface be denoted by the *x*- and *y*-axes. Figure 4 shows that the incident beam is propagated into the diffraction grating such that the first-order diffracted beam is directed toward the lens. For an accurate analysis, it is necessary to draw and analyze a complex 3D diagram, such as the angle of the incident beam and the alignment angle of the diffraction grating. Figure 4a shows that the diffracted beam propagates to the lens with ideal alignment of the diffraction grating. In this case, the diffracted beam is incident in alignment with the *x*-axis of the lens surface. When the diffraction grating rotates slightly around the *z*-axis, the direction of the grating grooves becomes *Ψ* with respect to the *x*-axis, and the aligned beam deviates from the *x*-axis center of the lens surface. However, if the groove direction of the diffraction grating in Figure 4b rotates slightly around the *x*-axis, it deviates completely from the *x*-axis of the lens surface, as shown in Figure 4c. 

The well-known diffraction grating equation is

(5) $$p\left(sin\;\beta +sin\;\alpha \right)=m\lambda$$

where α and β are the incident and diffracted angles, respectively, with respect to the normal axis of the diffraction grating; *m* is the order of the diffracted beam; *λ* is the optical wavelength; and *p* is the pitch of the grating [1,27]. When the groove direction of the diffraction grating and the *x*-axis are at an angle of *Ψ*, the aligned beam deviates from the *x*-axis of the lens. The diffraction grating equations are then given by

(6) $$p\left(sin\;x_{m}+sin\;x_{i}\right)=m\lambda \;sin\Psi$$

(7) $$p\left(sin\;y_{m}+sin\;y_{i}\right)=m\lambda \;cos\;\Psi$$

where *Ψ* is the angle between the direction of the grating grooves and the *x*-axis; $x_{m}$ and $x_{i}$ are the diffracted angle and incident angle for the *x*-axis of the grating, respectively; and $y_{m}$ and $y_{i}$ are the diffracted angle and incident angle for the *y*-axis of the grating, respectively [27]. If there is only a slight rotation about the *x*-axis of the grating, then the change occurring only in the *y*-direction, as expressed by Equation (7), can be considered. As the difference in $y_{m}\;$is very small, Equation (7) becomes

(8) $$p\Delta y_{m}=m\left(\Delta \lambda \right)cos\Psi ,$$

where $\Delta y_{m}$ is the difference between the diffracted angles when the shortest and longest wavelengths in the scanning wavelength range are aligned along the *x*-axis. We analyzed the output of the WSL for the three cases of diffraction grating alignment.

**Figure 5:**
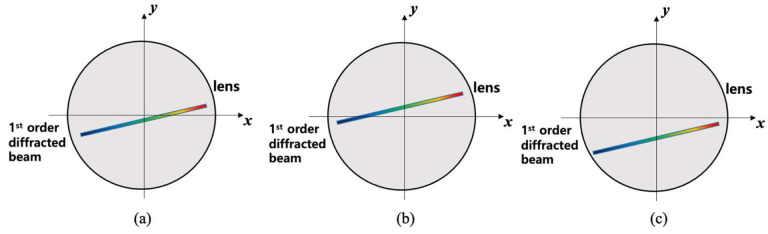
First-order diffracted beam incident on the lens surface according to the alignment state of the diffraction grating; (**a**) Case 1: initial state of the alignment, (**b**) Case 2: rotate −0.018° around the *x*-axis in Case 1, and (**c**) rotate 0.0045° around the *x*-axis in Case 1.

Figure 5a schematically shows the diffracted beam incident on the lens surface with the proper alignment of the diffraction grating. It can be seen that the directions of the grooves of the diffraction grating are out of ideal alignment with respect to the *x*-, *y*-, and *z*-axis (Case 1). When the alignment of the diffraction grating is adjusted by −0.018° by turning the knob in the *x*-axis direction, the position of the diffraction beam entering the lens surface changes, as shown in Figure 5b (Case 2). However, when the knob in the *x*-axis direction is adjusted to 0.0045°, the position of the diffracted beam entering the lens surface changes, as shown in Figure 5c (Case 3). 

**Figure 6:**
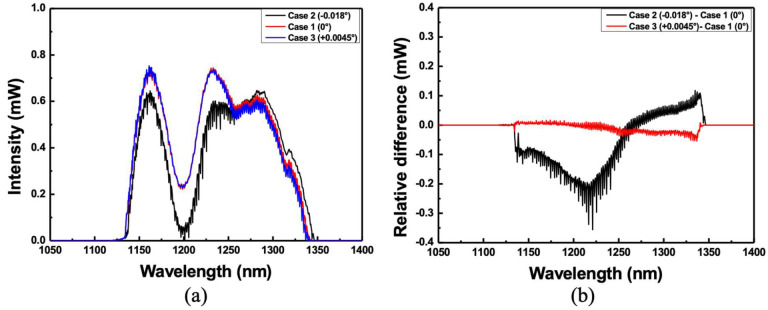
(**a**) Optical spectra on a linear scale for Figure 5, and (**b**) the difference between Case 2 and Case 3 based on Case 1.

Figure 6a shows the optical output spectra on a linear scale for each case in Figure 5, where the 10-dB bandwidth of the wavelength scanning for Case 1 is ~209 nm from 1131 nm to 1340 nm. Figure 6b shows the difference between Cases 2 and 3, based on Case 1. As the diffraction grating is slightly shifted with respect to the *x*-axis, the changes in the intensity of the short and long wavelengths differ in the wavelength scanning range.

**Figure 7:**
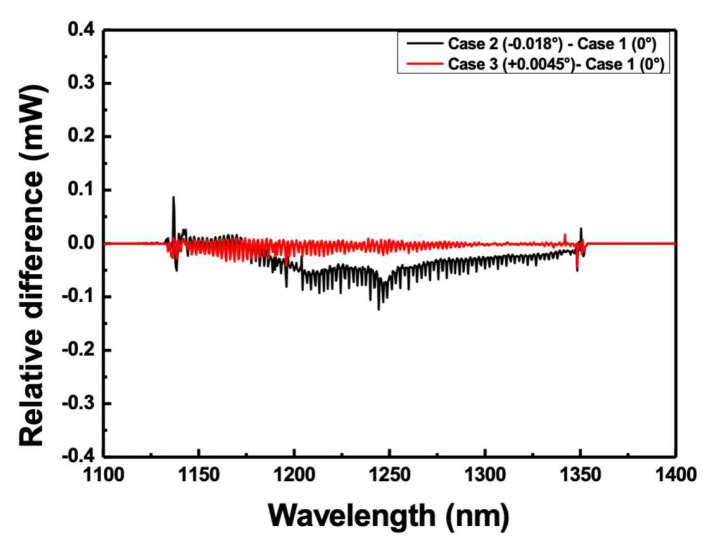
Results of reducing the error for $\Delta y_{m}$ after optimization of alignment.

By substituting the values of the variables used in the experiment into Equation (8) for m = 1, Δλ = 210 nm, p = 1/600 mm, and $\Delta y_{m}$ = 0.0225°, Ψ is calculated to be approximately 0.18°. It is difficult to measure small changes in the diffraction grating within 1°. When Ψ = 90° in Equation (7), both the 0th and 1st diffracted beams are mirror-reflected; therefore, $y_{0}=y_{1}$, that is, there is no difference in the angle of the *y*-axis [27]. However, if the direction of the grooves of the diffraction grating deviates slightly from Ψ = 90° with respect to the *x*-axis, then the diffracted beam is incident on the lens surface, as shown in Figure 5. By properly turning the knob to align the diffraction grating, the 0th and 1st diffracted beams can be aligned at the same point. Figure 7 shows the results of reducing the error for $\Delta y_{m}$. It is possible to reduce the difference in intensity in the scanning wavelength range, as shown in Figure 6b. Through the optimization of alignment, as described above, the wavelength scanning range could be further increased. The 10-dB bandwidth of the wavelength scanning obtained through alignment optimization is ~223 nm (from 1129 nm to 1352 nm) as shown in Figure 2b, which is greater by approximately 14 nm compared to the case in Figure 6. 

**Figure 8:**
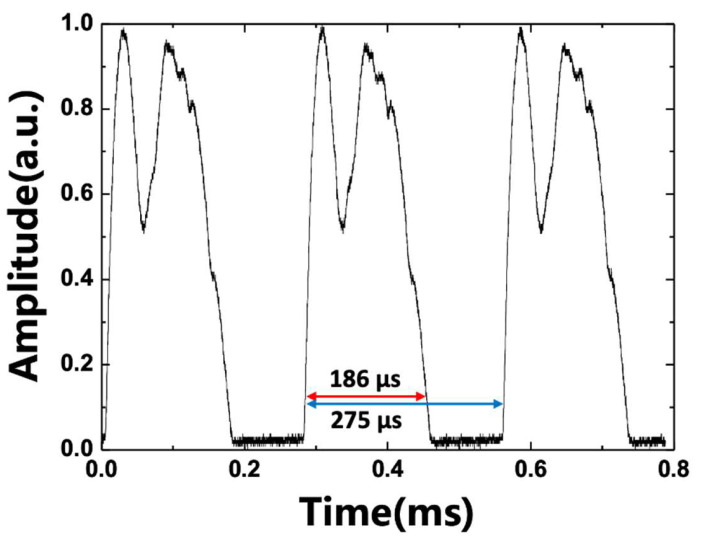
Temporal output from the wideband WSL.

In PMSWF-based WSLs, the FSR is related to the focal length of the lenses used in the filter and the spacing of the grooves of the diffraction grating, as described in Refs. [1,6,16]. In our experiments, the grating pitch p was $\frac{1}{600}\mathrm{mm}$; $\beta_{0}$ = 0.1 rad, which is the angle between the optical axis of the lenses and the normal axis of the diffraction grating; the focal length of the first lens *F*_1_ = 4.5 cm; the focal length of the second lens *F*_2_ = 10.0 cm; and the face-to-face polar angle of the polygonal mirror θ = $\frac{2\pi }{72}\;$rad. Therefore, the theoretically calculated ${\left(\Delta \lambda \right)}_{FSR}\;$is 321.6 nm. Figure 8 shows the WSL outputs in the temporal domain, where the FSR can be obtained using one-to-one correspondence between the spectral and temporal domain [16]. The wavelength scanning range of WSL is 223 nm in the spectral domain and 186 ms in the time domain, so one period in the spectral domain corresponds to ~329.7 nm for one period in the time domain of 275 ms. Therefore, the measured ${\left(\Delta \lambda \right)}_{FSR}\;$in the temporal domain is ~329.7 nm. The relative error between the measured and theoretical values is approximately 2.5%. This can be attributed to the uncertainty of the signal measurement in the temporal domain.

**Figure 9:**
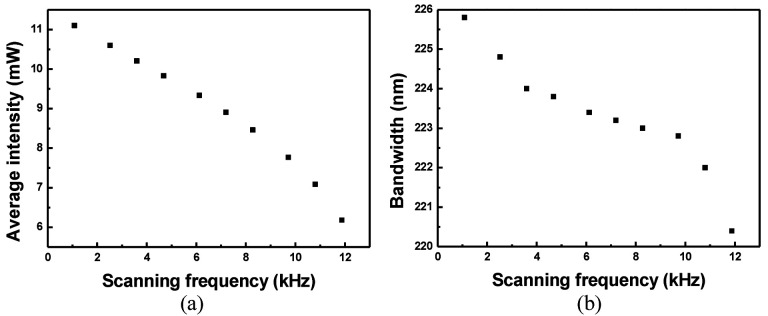
Output characteristics of wideband WSL according to the scanning frequency; (**a**) average intensity, and (**b**) scanning bandwidth.

The output of the WSL decreases in intensity and bandwidth as the scanning frequency increases [16]. Figure 9 shows a graph of the output characteristics of the wideband WSL according to the scanning frequency, up to 11.9 kHz. The average intensity gradually decreases from 11.1 mW to 5.3 mW with an increase in the scanning frequency, as shown in Figure 9a. However, the bandwidth gradually decreases from 226 nm to 223 nm until the scanning frequency is 10 kHz, and then shows a dramatic decrease above 10 kHz. 

**Figure 10:**
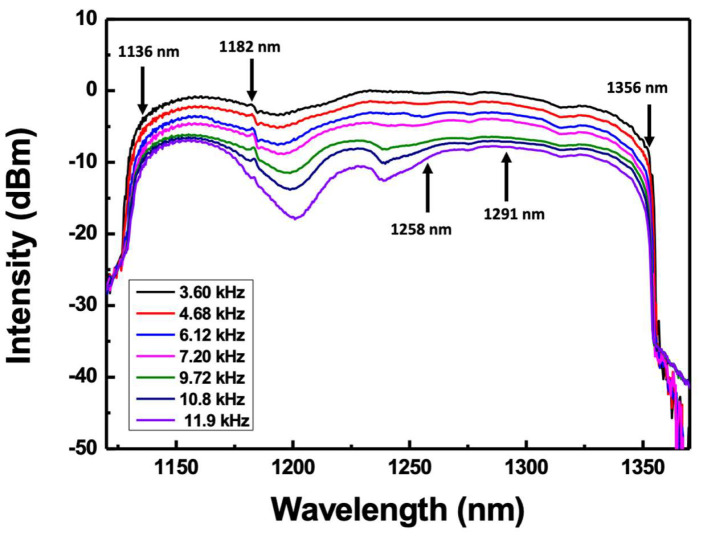
Scanning bandwidth variations of wideband WSL with scanning frequency.

The decrease in the bandwidth and average intensity as the scanning frequency increases can be quantitatively explained by the saturation limit frequency described in Section 2. We analyzed the saturation limit frequencies for several wavelengths. Figure 10 shows the variations in the spectral bandwidth from the wideband WSL according to the scanning frequency. Table 1 lists the small signal gain and loss per cycle at each SOA wavelength for the calculation of *β*. The gain and loss at 1136, 1182, 1258, 1291, and 1356 nm in the spectral range of Figure 10 were measured, as listed in Table 1. Additionally, by substituting the values in Table 1 and the variables for each wavelength in Equation (4), the saturation limit frequencies are obtained, as listed in Table 1. Here, the effective refractive index ($n_{ref}$) and the total tuning range ${\left(\Delta \lambda \right)}_{FSR}$ were 1.46 and 330 nm, respectively. The wavelength at 1356 nm is located at the edge of the scanning range, and 1182 nm is a wavelength with a large intensity change with increasing scanning speed. In addition, the wavelength of 1182 nm corresponds to the vicinity of the valley in the ASE spectrum, where the two SOAs are combined, as shown in Figure 2a. As shown in Table 1, the saturation frequencies of 1182 nm and 1356 nm are calculated to be 4.92 kHz and 0.41 kHz, respectively. However, for the other three wavelengths (1136, 1258, and 1291 nm), the saturation frequencies are calculated to be 9.94, 10.20, and 8.73 kHz, respectively. In the spectra of Figure 10, the intensity decreases faster near 1200 nm as the scanning frequency increases. This can be found in the ASE spectrum of SOA1 in Figure 2a. The ASE spectrum of SOA1 shows a weak relative intensity near 1200 nm. This can be seen as the result of insufficient gain recovery as the scanning frequency increases.

**Figure 11:**
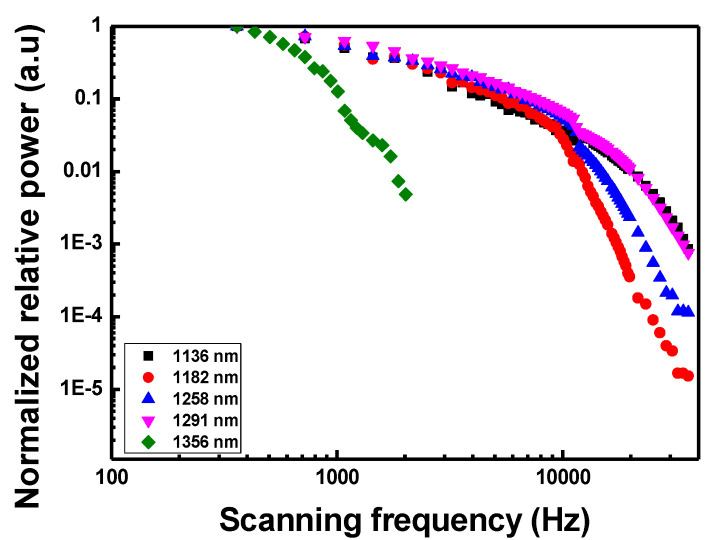
Relative intensity variations of each wavelength component as a function of scanning frequency.

Figure 11 shows the relative intensity variations of each wavelength component with increasing scanning frequency. Relative wavelength intensities were measured while increasing the scanning frequency by normalizing the intensity at 0.36 kHz to 1.0 kHz. The relative intensity at 1356 nm, located at the edge of the scanning range, decreases rapidly as the scanning frequency increases. The saturation limit frequency of this wavelength is less than 1 kHz, and as the scanning frequency increases, the scanning bandwidth decreases, as shown in Figure 9b. This is consistent with the fact that lasing is no longer possible above a certain frequency. Therefore, it can be seen that the decrease in the relative intensity according to the increase in the scanning frequency is slightly related to the saturation limit frequency. In addition, because the SOA has polarization dependence, an intensity difference according to polarization occurs over the entire scanning wavelength range and, thus, a relative decrease in intensity appears partially.

## 4. Conclusions

If a wavelength-swept laser (WSL) is implemented based on a single semiconductor optical amplifier (SOA), it is difficult to obtain a scanning range of 150 nm or more. However, if the WSL is implemented by combining two SOAs with different center wavelengths, it can have a scanning range of 200 nm or more. We successfully implemented a broadband WSL using a PMSWF by connecting two SOAs in parallel in the form of a Mach–Zehnder interferometer. The center wavelengths of each SOA were 1190 nm and 1285 nm, respectively, and the 10-dB scanning bandwidth of the WSL was 223 nm, from 1129 nm to 1352 nm. When the WSL scanning speed increased, the output intensity and bandwidth decreased. We analyzed these phenomena in terms of the concept of the saturation frequency limit that accumulates in the amplified spontaneous emission (ASE) background and reaches saturation power, and we experimentally determined that they are related in part. To increase the scanning range and output intensity in the WSL, a fine alignment of the polygonal mirrors and precise optical alignment between the two lenses and the beam incident on the diffraction grating are important. We were able to obtain the optimal optical output by analyzing the output dependence of the WSL according to the alignment of the optical axis of the diffraction grating and lenses. This broadband WSL is expected to be utilized as a light source for dynamic optical fiber sensors with a wide measurement range.

## Figures and Tables

**Table 1:** Small signal gain and loss per cycle and calculated frequencies of saturation limit at each wavelength from the broadband.

Wavelength l (nm)	Small Signal Gain (G) (dB)	Loss (dB)	*b*	$P_{sat}$ (dBm)	$P_{ASE}\;\;$(dBm)	$f_{max}\;(\mathrm{kHz})$
1136	27.10	22.80	2.69	5.87	−29.40	9.94
1182	18.10	14.00	2.54	8.00	−38.20	4.92
1258	24.40	17.47	4.93	1.98	−36.73	10.20
1291	24.50	18.36	4.11	5.82	−36.45	8.73
1356	24.94	14.77	1.04	−19.00	−47.36	0.41

## Data Availability

Data available on request from the authors.
